# A Case Study Perspective on Working with ProUCL and a State Environmental Agency in Determining Background Threshold Values

**DOI:** 10.3390/ijerph121012905

**Published:** 2015-10-15

**Authors:** David L. Daniel

**Affiliations:** Applied Statistics, New Mexico State University, Department 3CQ, Post Office Box 30001, Las Cruces, NM 88003, USA; E-Mail: ddaniel@nmsu.edu; Tel.: +1-575-646-4173; Fax: +1-575-646-1915

**Keywords:** environmental monitoring, expectation maximization (EM) algorithm, gamma distribution, maximum order statistic, non-detect, nonparametric, outlier detection, ProUCL, regression on order statistics (ROS), upper tolerance limit (UTL)

## Abstract

ProUCL is a software package made available by the Environmental Protection Agency (EPA) to provide environmental scientists with better tools with which to conduct statistical analyses. ProUCL has been in production for over ten years and is in its fifth major version. In time, it has included more sophisticated and appropriate analysis tools. However, there is still substantial criticism of it among statisticians for its various omissions and even its philosophical approach. Due to limited resources, some state agencies have set ProUCL as a standard by which all state-mandated environmental analyses are compared, despite the EPA’s more open acceptance of other software products and methodologies. As such, it can be difficult for state-supervised sites to convince the state to allow the use of more appropriate methodologies or different software. In the current case study, several such instances arose and substantial resources were invested to demonstrate the appropriateness of alternative methodologies, sometimes without acquiring acceptance by the state despite sound statistical demonstration. In particular, efforts were made to address: inappropriate outlier detection, upper tolerance limit (UTL) calculations based on gamma distributions when non-detects were present, and inappropriate use of nonparametric UTL formulas.

## 1. Introduction

### 1.1. Mandated Soil Background Threshold Limits

In 2012, a work site (the site) was instructed by the governing state agency to develop a background soils investigation plan for the purpose of calculating various descriptive statistics and background threshold values (BTVs) at various depths, for several soil types found at the site, and for 34 analytes (substances whose chemical properties, such as concentration, are of interest). A BTV is generally calculated from sources nearby a monitored site and used to assess whether concentrations of analytes at the monitored site are high by comparison. This assessment is often conducted on an ongoing basis over time, so the calculated BTVs could serve as reference levels for many years. The developed plan called for collecting a random sample of *n* = 12 observations from five soil types found in the area and at three depths, and that separate BTVs would be calculated for each of the 510 combinations (five soils × 3 depths × 34 analytes) based on upper tolerance limits (UTLs). A UTL is a numeric value calculated from the sample data in a manner such that it will exceed a specified percent of the population from which the sample was selected-known as the coverage, with a specified level of confidence. In this scenario, the coverage was specified to be 95% and the confidence level was to be 95%, as well. These calculations were to use appropriate distribution-specific UTL formulas depending on whether distribution tests indicated the samples reasonably appeared to come from normal, lognormal, or gamma distributions, or to use a nonparametric UTL formula if no distributions appeared to be appropriate. The detection limit (DL) is the lowest concentration of a chemical that a particular measurement machine is capable of reliably detecting. In environmental sampling, it is not uncommon for concentration levels to be below the DL, thus resulting in a non-detect (ND) value and causing the data to be left-censored. An ND simply indicates that the concentration present in the sample is between zero and the DL. Hence, the presence of NDs provides limited but valuable information about the analyte population of concentrations. However, due to lacking specific quantitative values, using NDs in statistical analyses is complicated and there is rarely a single optimal mechanism for working with them. Early recommendations were often to replace the ND with half of the DL (DL/2), but this often causes a frequency spike at that value. In reality, one would expect the unknown true values for the NDs to be spread as per the underlying distribution, so using DL/2 in place of NDs often misconstrues results in statistical calculations.

### 1.2. ProUCL Software

ProUCL [1] is a software package made available by the US Environmental Protection Agency’s (EPA’s) Office of Research and Development (ORD) to provide researchers working with environmental data with better tools with which to conduct statistical analyses. The EPA has contracted out the development of ProUCL to Lockheed Martin since early on in its evolution. ProUCL runs only under the Windows operating system. Its development began in 1999 for internal EPA use and grew into a publicly released software package. It is now at Version 5.0.00, which was released in September 2013, and comes with both an extensive User Guide [1] and Technical Guide [2]. The latter half of its name reflects its early focus on upper confidence limit calculations, but in time, it has evolved to include much more, including one and two sample hypothesis testing, ANOVA, regression, trend analysis, outlier detection, goodness of fit testing, and graphical methods, and often provides both parametric and nonparametric methods. However, what distinguishes ProUCL from many other software packages is its emphasis on statistical interval calculations (such as upper confidence limits (UCLs), upper prediction limits (UPLs), and UTLs [3]), its ability to work with left-censored data, and its availability of methods for distributional assumptions often associated with environmental data (lognormal, gamma, and nonparametric). In time, it has incorporated more sophisticated and appropriate analysis tools, particularly with regard to handling data containing NDs and data following a gamma distribution.

Despite its substantial evolution, there is still criticism of ProUCL among statisticians for its various omissions and even its philosophical approach. One area it has been criticized for is its lack of spatial analysis capabilities to account for the spatial correlation that is often present in environmental data. However, considering ProUCL’s primary audience, incorporation of sophisticated spatial analysis methodologies may not be a reasonable consideration. Other criticisms include the lack of specific methodologies relevant to environmental analyses, such as techniques that utilize Expectation Maximization (EM) and Monte Carlo Markov Chain (MCMC) methods for handling NDs, and more advanced outlier detection methods. The ProUCL Version 5.0.00 User Guide states that, while it provides many methods that are described in EPA documents, “one may not compare the availability of methods in ProUCL 5.0 with methods available in the commercial software packages such as SAS and Minitab 16,” and suggests the use of commercial software if methods outside its scope are required, specifically mentioning tests that correct for seasonal/spatial variations. For more robust outlier techniques, the ProUCL User Guide encourages interested users to try the Scout 2008 software package [4]. Additionally, ProUCL has a limiting interface that lacks a programming language which consequently prevents automation of repeated processes, makes it difficult to export or extract specific result outputs from analyses performed by groups, and precludes the conducting of simulations for comparison with other software or between various statistical methods (both those methods available in ProUCL and those not included).

### 1.3. Analysis and Reporting to the State Agency

As the site’s statistical consultant, my role is to perform the agreed upon analyses, document statistical results in a written report (which is generally included in the site’s report to the state agency), and help the site reply to the state agency’s response to their report. The state agency can either respond to the site’s report with a notice of approval, a notice of approval with modifications, or a notice of disapproval. The latter two notices are accompanied by a list of issues that need to be addressed. In this scenario, my client’s first two reports were each met with notices of disapproval over statistical concerns. The third report submission was responded to with a notice of approval with modifications.

Most of the issues that were the state agency’s grounds for disapproval were due to the agency’s lack of understanding of statistical issues, its reliance on the ProUCL software and the ProUCL documentation for guidance, and—at a more fundamental level—its general lack of resources. The remainder of this paper discusses the main statistical issues over which the state agency had objections and what was done to address these objections. I then present my perceptions of how a lack of state resources, the state agency’s policies, and the EPA’s provision of ProUCL were each factors leading to the objections, followed by some recommendations for the future.

## 2. Statistical Issues

### 2.1. Outlier Detection

Going forward, it is important to note that lognormal and gamma distributions are commonly used to model environmental chemical concentrations. In the present scenario, of the samples viable for analysis (some are excluded from analysis due to excessive NDs), based on goodness of fit (GOF) tests, 70.0% were associated with gamma distributions and 30.0% were not associated with any distribution considered, while none were associated with a lognormal distribution.

The first notice of disapproval from the state agency expressed concern that most of the calculated UTLs were greater than the maximum value of the sample from which each UTL was calculated. The state agency expressed concern over this because they believed this was not typical. While the agency did not explain why this comparison was made, it is not uncommon for them to use the maximum as the BTV when a better value is not available. This, however, is indeed typical with samples of size twelve—A short simulation (five lines of R code [5]) shows that more than 99% of the time a calculated UTL will exceed the maximum when a sample of *n* = 12 is drawn from a lognormal distribution or from a variety of gamma distributions. The notice went on to suggest that this unusually large number of occurrences of UTLs exceeding the sample maximum could be the result of unaccounted for bias, possibly due to outliers. Consequently, it requested a discussion of outlier detection and whether outliers were excluded in the study. While it is true that an outlier would increase the UTL, it would also increase the maximum sample value. In fact, another short simulation shows that increasing the maximum sample value in a sample (so as to mimic an outlier) taken from either a lognormal distribution or a variety of gamma distributions causes a decrease in the relative occurrence of the UTL exceeding the sample maximum. Since it is the comparison of these two values that the agency was using as an indicator of a problem, it is not reasonable to conclude that outliers may be the cause of this phenomenon. Even so, the site’s response needed to address the issue of outliers.

In statistics, identifying outliers is a tricky business as the definition of an outlier depends on the underlying distribution, yet identification of the underlying distribution generally depends substantially on the sample data that, if it contains outliers, will misrepresent the distribution that needs to be identified. The usual objective of outlier detection is to determine if a data point has been corrupted or if there is more than one population that has been included in the sample. Both the lognormal and gamma distributions are right-tailed distributions, which implies that they are prone to occasionally having a high value in a random sample, and these are quite often identified as outliers in outlier detection methodologies despite the fact that they do come from the same distribution as the other sample data. Sometimes an outlier can be identified as coming from an experimental unit that was problematic (e.g., there was difficulty controlling the temperature of a water tank used to grow algae in a biofuel experiment) or it may be clear that there was a data entry error. In such cases it may be reasonable to exclude or adjust the observation. However, it is a generally good statistical principle that, lacking other relevant information about an outlier, observations are not excluded since they may represent important information about the population being sampled (as often occurs with lognormal and gamma distributions).

In my reply to the request for a discussion of outliers in the study, I presented rationale similar to that given above for why removal of outliers was generally not a good idea and supported it with statistical references—Including an official EPA guidance document. I also informed them that my client conducted a data check among the most extreme values and found no reason to believe there were problems with the data collection or the data entry. Lastly, I developed simulations to demonstrate the ineffectiveness of commonly available outlier detection methods with right-tailed distributions. In particular, the ProUCL software—Which the state agency was strongly encouraging us to use (and eventually insisting that we use for some analyses)—Contains only two outlier detection methods—Dixon’s test and Rosner’s test. The former is generally used for samples with *n* ≤ 25, while the latter is used for *n* > 25. However, both tests assume a normal distribution for the underlying population (excluding the outliers). In the ProUCL Technical Guide, the authors state that these two methods have shortcomings (*i.e.*, masking effects, meaning that they tend not to identify all of the outliers), and refer the reader to more effective robust outlier identification procedures that they state “are beyond the scope of ProUCL 5.0.” They point the interested user to other resources, of which a primary technique is the PROP robust method [6]. However, like the other two tests, the PROP method also assumes normality. None-the-less, in order to demonstrate to the agency the lack in capability of the available outlier detection methodologies suggested by the ProUCL guidance, I conducted some simulations. Simulations were performed using both Dixon’s test (since our study had *n* ≤ 25) and the PROP method for samples of *n* = 12 from several gamma distributions that appeared to be typical of our study samples’ distributions based on GOF tests and parameter estimates. Dixon’s test falsely identified outliers between 15.7% and 38.1% of the time, depending on the distribution used. The PROP method falsely identified what its authors call “clear outliers” 68.7% to 88.7% of the time, and detected additional samples as having what the authors call “potential outliers.” Note that these results are no indication of the abilities of these two outlier detection methods under their specified assumption of normality. Rather, it only shows they have a high false positive rate under the much more common reality where the distributions of environmental concentrations tend to be more skewed.

These efforts arrested further criticism and inquiry from the state agency regarding outliers. Still, considerable expense and resources went into addressing these concerns that were brought about because of a lack of understanding by the state agency regarding UTL calculations, the impact of outliers, and outlier detection methods and principles.

### 2.2. Estimation of Distribution Parameters in the Presence of NDs

By far, the most contested issue in the soil investigation was over the method by which gamma distribution parameters were estimated when NDs were present in the sample. In order to calculate a UTL from any of the numerous samples whose distributions were associated with a gamma distribution, any NDs present in the sample had to be replaced by reasonable values. A method that is available in ProUCL is regression on order statistics (ROS). ROS takes advantage of the consistent proportionality of the spacing in the expected values of normal data, which *does not* depend on the parameters of the normal distribution (*i.e.*, the mean and variance). ROS has two phases. First, a straight-line regression equation is obtained for the *detected* sample values *versus* their corresponding expected values for the standard normal distribution, and second, the expected values of the standard normal distribution corresponding to the (generally smaller) NDs are plugged into the regression equation to obtain estimates for the ND values. These regression estimates can then be used as replacement values for the NDs in order to calculate the UTL.

The ROS method was initially proposed in the site’s soil investigation plan as a method for replacing NDs in samples. This method works well for normal or lognormal data, but as I began to read about how this was implemented in ProUCL for gamma distributions, I came to the conclusion that it was very naive in its estimation method. In particular, unlike using ROS for normal and lognormal data, the gamma ROS first requires estimation of the underlying distribution’s parameters because the proportionality of the expected values *does* depend on these parameters. The ProUCL implementation of ROS for gamma distributions first estimates the gamma parameters (as is necessary), and then applies the usual ROS method as described above, but using expected values from the gamma distribution having the estimated parameters. However, in estimating the gamma parameters, not having the (generally lower) values to substitute for the NDs will result in biased parameter estimates. This in turn gives biased substitution values for the NDs in the second phase. This method can be improved substantially by implementing a well-documented statistical technique known as the Expectation Maximization (EM) algorithm [7,8], which would iterate multiple times between estimating the gamma parameters using maximum likelihood estimation (MLE) and estimating the ND substitution values, eventually converging to a stable set of gamma parameter estimates and ND substitution values. This method is not available in the ProUCL software. ProUCL does provide another methodology for calculating a UTL for gamma-distributed data with NDs. This method obtains Kaplan-Meyer (KM) estimates of the mean and standard deviation that do not rely on any knowledge of the underlying distribution (*i.e.*, they are nonparametric estimates), and then plugs these values into a UTL formula that is specific to the gamma distribution. For the calculation of UTLs for samples conforming to a gamma distribution, I opted to use the EM approach for two reasons: (1) it utilized the ROS methodology, which the original investigation plan called for (and the state agency specifically recommended that ROS be used in a formal response to the site’s initial soil study plan); and (2) I was less familiar with the capabilities/performance of the KM approach.

In the first notice of disapproval from the state agency, concern was expressed over the magnitude of the UTL estimates. As noted in the previous subsection, the agency was concerned that outliers might be the cause of the large magnitudes, but they were also concerned over the methodology being used and the fact that it was not a method implemented by ProUCL. In my response, I showed results of simulations I conducted that demonstrate the EM algorithm provided better estimates than the gamma ROS implementation of ProUCL and made all of my code available to the agency-both for calculating the UTLs via the EM method and for conducting the simulations. Simulation results specific to the case study scenario and very similar to those presented to the state agency are presented in this paper’s Appendix. Note that the results only address the EM method’s capabilities under the study’s typical conditions of *n* = 12, having a single DL, and having an average of three NDs per sample. However, subsequently, in the second notice of disapproval, the agency expressed concern over the difficulty involved in reviewing my code-not because it was disorganized or poorly commented, but because I had modified an existing function in the R library to implement the EM algorithm. In fact, the notice stated regarding the site’s response that, “While the response included a detailed discussion of how the statistical analyses were performed, there are still some concerns with the upper tolerance limits.” Trying to determine exactly what those concerns were or how they could be addressed was difficult, but hints were given by their later statement that the “modifications appear to stray from EPA’s (and (the state agency’s)) preferred/recommended statistical package for environmental applications—ProUCL”. However, this was exactly the point, which had been clearly stated in the original report—That is, the implementation of the gamma ROS method in ProUCL was a poor methodology for our study scenario, and simulations had been provided to demonstrate this, along with the simulation code.

Further insight is gained by the state agency’s statement that, documentation for the R software used “would need to be compared to the recommendations in the ProUCL documentation to ensure they are similar or that the R software and modules represent a more conservative approach”. This statement conveys two important implications. First, the agency is relying heavily on the ProUCL documentation of its methodologies for guidance, even when the argument being made is that the ProUCL methodology is poor, and second, in the context of other statements made by the state agency, from the agency’s perspective, the notion of being a “more conservative approach” means that it yields UTL values that are as small or smaller than those given by ProUCL methods. This can be seen in the agency’s later statement that if the site wished to use the UTL calculations obtained via the EM method, the most straightforward approach would be “to demonstrate that the approach taken is similar to and conservative compared to ProUCL.” Ultimately, the agency requested that we examine and eventually use the KM method available in ProUCL for many of the UTL calculations. Due to funding and time limitations, I did not have an opportunity to investigate the KM method as thoroughly as I would have liked and, in particular, was unable to conduct simulations to compare it to the EM method. Based on what I have read about the KM method, I have no general qualms in using it, as I did with ProUCL’s gamma ROS method. What is disconcerting is the rationale for using it as, in the end, the agency specifically requested that the site use the method that gave the smallest UTL calculations, giving no consideration to which method was best from a statistical perspective.

### 2.3. Use of Nonparametric UTL Formulas

GOF tests (along with various graphical methods) are generally used to ascertain if a distribution type can reasonably describe the population from which a sample was obtained. In situations where no distribution can be reasonably assumed to describe the underlying population, a category of methods known as nonparametrics may be useful. By definition, nonparametric methods do not rely on the shape or other properties of any particular distribution type, but are generally based on mathematics that rely on the probabilities of the various possible combinations that might be obtained. It is important to realize that nonparametric methods typically are not free of assumptions or conditions, and are often used inappropriately without reasonable verification of the assumptions and conditions. Nonparametric UTL formulas are no exception and have minimum sample size conditions in order to obtain the specified level of confidence and coverage.

When the site began developing the study plan in 2012, the most recent incarnation of ProUCL was Version 4.1.00, and the site project team utilized the corresponding ProUCL Technical Guide [9] in developing an analysis strategy that provided different methods of computing UTLs depending on the presence and quantity of NDs, the sample size, and the ability to identify a suitable distribution for the underlying population. The introduction of the ProUCL Version 4.1.00 Technical Guide discusses the difficulty of assessing the underlying distribution when NDs are present, and then states, “In such situations, it is preferable to use nonparametric (e.g., KM method) methods to compute statistics of interest such as UCLs, UPLs, and UTLs. Nonparametric methods do not require any distributional assumptions about the data sets under investigation. Singh, Maichle, and Lee [10] also concluded that the performance of the KM estimation method is better (in terms of coverage probabilities) than various other parametric estimation (e.g., MLE, EM, ROS) methods.” This led the site project team to believe that the referenced KM method was nonparametric, and thus to develop an analysis strategy that stated KM methods for calculating UTLs would be used under certain situations where an underlying distribution could not be satisfactorily identified. After the plan was approved by the state agency and I began to read more about implementing the KM methods for calculating UTLs, I came to the realization that these methods are not actually nonparametric as the above statement in the ProUCL documentation directly implies. Rather, while the KM estimations of the population mean and standard deviation are nonparametric, the UTL calculations referred to in this statement merely utilize these KM estimates in various parametric-based equations, thus still necessitating reasonable identification of the underlying population. Additionally, I also discovered that the cited paper by Singh, Maichle, and Lee [10], concluding that the KM method was better only pertained to UCL calculations, and the paper does not attempt to address UTLs.

This caused a conundrum in the analysis process since the investigation plan called for a nonparametric method (and the state agency was expecting one), yet the KM methods were not appropriate as they are not actually nonparametric. Further, the usual nonparametric method utilizing maximum order statistics requires minimum sample sizes of *n* = 59 to achieve 95% confidence in a UTL with 95% coverage. Our sample of *n* = 12 would only provide 95% confidence for a coverage of 77.9% of the population, which defeats the purpose of calculating a UTL to be used as a BTV. To address this issue in an ad hoc manner, I used a nonparametric method that used the KM calculations of the mean and standard deviation in Chebyshev’s formula (ProUCL Technical Guide v. 5.0.00, Section 3.5.4.1) to calculate a 95% Upper Prediction Limit (UPL) for a single future observation. A UPL is a numeric value calculated from the sample data in a manner such that it will exceed a specified number of future observations from the population from which the sample was selected with a specified level of confidence. This UPL was then bootstrapped (randomly selecting twelve observations with replacement from the original sample of twelve observations and calculating the UPL with the resampled values, and repeating this many times) and the 95^th^ percentile of 1000 bootstrapped UPL values was taken as an approximate UTL.

Some simulations bootstrapping a gamma-based UPL in a similar manner (for samples of *n* = 12 without NDs from various gamma distributions) resulted in UTL approximations that ranged from 0% to 10% under the usual gamma UTL formulation (based on the Wilson–Hilferty approximation [11]). As the Chebyshev inequality is conservative, it will give larger estimates, and simulations bootstrapping a Chebyshev-based UPL resulted in UTL approximations that ranged from 17% to 40% above the usual gamma UTL formulation. This implementation of the Chebyshev UPL to get an approximate UTL resulted in UTL values that were often two times larger than the sample maximum, and occasionally approaching three times as large. This ratio of the UTL estimate to the sample maximum generated great concern from the state agency. However, ProUCL does implement the Chebyshev UPL formula as a nonparametric UPL calculation, though it does state that for larger skewed data sets the user may want to compute the Chebyshev UPL using a confidence level of 85% or 90% rather than 95%. Some simulations implementing the Chebyshev UPL calculation as per ProUCL guidance (for gamma distributed data with *n* = 12) resulted in Chebyshev UPLs exceeding UPLs from the gamma-based formula by 25%–75%. In light of this, the 17%–40% exceedance for the UTL estimates does not seem unreasonable. In the last response from the state agency, which was a notice of approval with modifications, the agency did not even address the mechanism for calculating UTLs in this situation, *i.e.*, when no distribution can be reasonably assumed to describe the underlying population, thus apparently leaving this to the discretion of the site.

It should be noted that the section in the ProUCL Version 4.1.00 Technical Guide containing the above noted statement that led to the inappropriate specification of the “KM method” has been substantially rewritten in Version 5.0.00, and now more clearly describes the available methods. In fairness, documenting the methods available for such a broad combination of situations is complex and the effort required is, no doubt, substantial. In total, the ProUCL Version 5.0.00 Technical Guide is an impressive document with lots of useful information, often accompanied by relevant technical details.

## 3. Discussion

### 3.1. The Role of the State

For the three issues with the state agency discussed in the previous section, what was most concerning was (1) the reliance the agency had on the ProUCL software and its documentation; (2) the agency’s inability to independently assess the statistical methodologies used to perform the statistical analyses; and (3) the fact that its criterion for selecting the UTL method to be used was based on which method gave the lowest UTL, regardless of the statistical evidence behind the methodologies to be considered.

Unfortunately, the three issues discussed in the previous section for the current scenario are not isolated issues. Additionally, in the first notice of disapproval, the state agency misinterpreted the implication of high p-values in the GOF tests, stating that, “it appears that even if the p-values were greater than 10%, when nonparametric distributions may be applied, the data were still forced into one of the four distributions.” In fact, when the p-value for a GOF test was greater than α = 10%, it indicated failure to reject the null hypothesis that the sample data came from the tested distribution, which would mean we did not have evidence against the appropriateness of that distribution. Hence, we were not forcing a distribution on a data set, but were adhering to fairly standard statistical practices for determining the appropriate distribution using GOF tests. The agency’s concern would have been valid only if the *p*-values had been low, not high.

Additionally, in a previous study to compare up-gradient ground water to down-gradient ground water, the site called me because the state agency wanted an early assessment when the site had only collected samples of *n_u_* = 5 and *n_d_* = 1, respectively. In this case, the state agency gave the site a formula for a two-sample t-test it wanted the site to use to assess whether a difference existed in the up-gradient and down-gradient population mean concentrations, but the formula completely omitted a variance term in the denominator for the down-gradient sample. Most likely this was because the agency adapted a two-sample t-statistic from an EPA guidance document but, being unable to calculate the sample variance for the down-gradient sample since *n_d_* = 1, they simply dropped the variance term from the formula. This had the effect of inappropriately magnifying the *t*-value by a factor of approximately 2.45 (assuming the variances are reasonably similar in the two populations), thus magnifying the estimated difference between the population means relative to the estimated variability in their differences due simply to random error, and greatly increasing the likelihood of rejecting the null hypothesis of no difference in the population means.

In yet another study, the site called me on three different occasions to do fairly substantial statistical analyses on the same data with the same analysis objective—Each time using a different methodology (each dictated by the state agency) because the state agency changed its mind twice about how it wanted the data analyzed.

The take away point is that the state agency is charged with overseeing processes that it does not have the statistical expertise to oversee and either does not have the resources to contract it out or is not willing to do so. In the scenario discussed in the previous section, the state agency even hired an external individual that it referred to as a “risk assessment subcontractor” to review the statistical analyses performed, yet this individual also did not have adequate expertise. Some of the statistical issues the agency deals with are not very complicated, but many of them are quite complicated. These complicated issues simply cannot be assessed without substantial statistical expertise and will often, at the very least, necessitate the involvement of an individual with a Masters degree in statistics and substantial relevant experience, and in some cases will absolutely require the expertise of someone having a Ph.D. in statistics. As is demonstrated by the interactions with the state agency illustrated in the previous section, the state agency’s failure to acquire such expertise often costs their supervised sites substantial resources in attempting to alleviate concerns from the state agency. If the state had adequate statistical expertise, many of its concerns would be addressed internally and never even be presented to the sites. In the end, a site may not even be able to convince the state agency to implement good statistical practices even when the site employs a Ph.D. statistician because the state agency is not able to assess the statistical work.

A question that may arise in the mind of the reader is whether good communication between the state and the site could help alleviate the consequences of the agency’s lack of statistical expertise. The answer is-very likely, yes. Unfortunately, the state agency is extremely reluctant to allow direct communication between external subcontractors. In the current scenario, the site requested a meeting with the agency’s risk assessment subcontractor so that we could communicate interactively and clarify some issues, but the state agency flatly turned down the request. In fact, it has been my experience that the state agency’s employees are very reluctant to talk with statisticians. On the one occasion, I was able to arrange a conference call (many years ago), the two individuals on the call from the state agency were exceptionally hesitant. This is not an uncommon scenario when there is an imbalance of knowledge, and the only solution is to increase the expertise within the state agency. To be more pointed, states have an ethical responsibility to ensure that their agencies acquire and maintain adequate expertise to perform the duties with which they are charged. Statistics is a complicated field and environmental monitoring scenarios frequently encounter complex statistical issues. Often only a Ph.D. statistician can adequately address these issues. Not having such expertise available when needed in areas such as environmental monitoring is costly to the state’s businesses and risky for the state’s residents.

### 3.2. The Role of the EPA

When I think about the broad goal of the EPA—to protect human health and the environment—I am overwhelmed with how tremendous a task this is. One major role of the EPA is to develop guidance for states to follow, and these are set forth in a variety of documents that are revised as new advances are made. I have witnessed the evolution of some of these guidance documents and they have improved substantially over the years. However, the EPA also does site assessments, and in speaking with the Director of the Site Characterization and Monitoring Technical Support Center (SCMTSC), which is responsible for supporting and developing the ProUCL software, I was told that the EPA will assess statistical analyses performed using any software product. Of course, the EPA has much more depth and expertise than any state agency, and this is partly the price states pay for having autonomy in overseeing the protection of their own state’s environment. While the EPA provides guidance to states, it does not generally dictate how states implement processes to protect their environments. While such liberty has its appeal, it can often result in poor implementations. While working with a site to develop a particular assessment plan some years ago, I contacted the governing agencies for six different states and inquired how they generally analyzed the data obtained from such an assessment plan. While a couple of the analysis strategies were similar, there was substantial variation in them, and there was only one analysis strategy of the six that I thought had merit, and even it was lacking.

With such autonomy given to the states, it is difficult to make recommendations as to what more the EPA could do to improve the capabilities at the state level. It is clear that states would benefit from having greater statistical expertise available; so one recommendation to the EPA would be that it strongly encourages acquiring such expertise. In fact, the ProUCL Version 5.0.00 Technical Guide states that a project team may want to consult with a statistician nine different times throughout. Yet this is clearly not adequate encouragement for many state agencies. I believe seeking expertise could be further encouraged by incorporating statements at several places in the ProUCL documentation that (1) the methodologies it provides are not all-inclusive; (2) some of the methodologies may not be appropriate or optimal in some situations; and (3) some situations may necessitate consultation with a statistician who can provide other insights and useful methods. Such language would have allowed me to point the state agency away from ProUCL as their definitive standard and encourage them to rely on the expertise of another consulting statistician.

The ProUCL software has progressed steadily since its inception, and has a substantive set of methodologies available in it. As a statistician, I have found its Technical Guide to be a valuable resource for learning about useful methodologies in environmental monitoring. I have also contacted the director of the EPA’s SCMTSC and requested copies of the Technical Review Forms that were completed by a number of users for version 5.0.00 of ProUCL. I was reasonably impressed with the effort this office made to solicit critique of the software from users of ProUCL and the replies made in response to the reviews. However, in reading the reviews it was clear that almost none of the reviewers had much statistical expertise, as this could be easily inferred by the gaps in knowledge in one or more statements made by almost every reviewer. It is difficult to believe that such a review process is very comprehensive when the software contains complex statistical methodologies yet is not reviewed by statisticians with advanced degrees. It would be my recommendation that the EPA’s SCMTSC specifically engage statisticians with advanced degrees in the reviews of ProUCL. As ProUCL is reasonably large at this point and, to my knowledge, has not undergone such a comprehensive review, initial reviews of the various components of the software should each be delegated to a few statisticians.

The interface to ProUCL is limited by a few licensed technologies that the authors have utilized to reduce some of the programming necessary (e.g., to import data and export analysis results). These technologies are less than optimal and make working with ProUCL more difficult than it otherwise would be with better technologies. In particular, extracting specific results is not well facilitated when numerous analyses are conducted “by group”. This was also reflected in multiple comments in the user reviews that I read.

ProUCL does not allow much automation because it lacks any type of programmatic interface. A comprehensive programming language would be useful for several reasons, including allowing more control over output and extraction of results, permitting simulations to allow comparison among various methods (both those included in ProUCL and others that are not), and automation of repeated analyses. However, the incorporation of a comprehensive programming language would be a major task and very unlikely to be funded. Because of this, and for other reasons outlined below, I contacted the director of the EPA’s SCMTSC and asked if the source code for ProUCL was available to the public. The director stated that it had never been requested before, but that such a request could be considered if there were specific reasons for it. Hence, I emailed the director with an outline of points as to why I believe the community would benefit from making the source code available. An abbreviated version of those points is included here:

1. It would facilitate side-by-side comparisons of methodologies via simulations and ensure that the methodologies used in the simulations faithfully represent those in the ProUCL software.
2. It would facilitate extraction of specific results from a large number of analyses. In particular, once the code was incorporated into programming environments, such as R or Python, a user could quickly extract just the outputs that are needed for a report.
3. It would allow users to better understand how calculations are performed where the manuals are lacking explanation. Having the code available would allow a sophisticated user to study the implementation and determine precisely what is being done in the ProUCL implementation of a particular method.
4. It would improve analysis workflow, as having the code available would allow a user to implement analysis processes that could be replicated or modified/updated with minimal effort.
5. It would promote the philosophy of reproducible research, which has gained tremendous momentum in the past couple of years. This would be accomplished by incorporating the code into the many software packages where reproducible research tools are already available. It is also very difficult to describe an analysis process that was followed for a large analysis when performed via a graphical interface, and a programmatic interface is generally necessary to ensure the analysis workflow is reproducible.
6. It would reduce the local environmental agencies’ views of ProUCL being the sole standard for comparison. Having the code available would eventually result in its implementation in other environments, and thus ultimately push local environmental agencies to more readily consider analyses performed in other software environments.
7. It would allow implementation of the ProUCL methodologies on non-Windows operating systems without having to run via virtual machines. Many software packages used for analysis, such as R and Python, are implemented across most platforms.
8. It would allow the statistics community to contribute code for consideration for use in ProUCL. With the code available, statisticians would be encouraged to investigate it and compare existing ProUCL methodologies to other methodologies, including new ideas for future methodologies. This will also lead to the development of more extensive guidelines as to when various methodologies should be used.
9. It would lead to faster fixing of bugs and general difficulties in ProUCL and its documentation. Any major software product has bugs, and having other statisticians evaluating and using its code base would lead to quicker identification of bugs and fixes, ultimately improving its reliability.

When I recently contacted the director about the status of this request, I was told that it would be discussed with the project support team for consideration and that it would be necessary to check on the proprietary issues.

### 3.3. The Role of the Statistician

Statisticians have responsibilities in their role as well. When a client of mine has been tasked by a state agency to perform an analysis using specific methodology that I believe is inappropriate or less than ideal, there is no doubt that it is going to be implemented by my client regardless of whether I perform the specified analysis or not, since they are under contract with the state agency. However, it has been my policy that if I am going to participate, I will do what the client has been tasked to do, but also implement a methodology that I deem to be appropriate for the analysis goals. Then, in my report, I present both sets of results and I attempt to make the case for why the mandated method is inappropriate/inferior while the newly proposed method is appropriate/superior. I have been fortunate that my clients have been open to affording me the resources to do this, and I believe that it has enhanced our relationship because they perceive it is a beneficial approach-accomplishing the contracted work while educating the state agency. Continuing to educate researchers in other fields about statistical principles, methodologies, and complexities should be a goal for every statistician, and we should seek opportunities to do so.

Additionally, I am always learning more about the areas in which I consult (such as environmental monitoring, though I consult in many areas), and hopefully learning better ways to do things as a consequence. For example, while I have always expressed to the site that continuing to take background samples over time can help refine the UTL calculations, in the future I will likely try to encourage that this be written into the investigation plan as part of the data quality objectives (DQOs). This would allow adapting to the lack of statistical techniques available when a reasonable underlying distribution cannot be identified and the sample sizes are too small to implement available nonparametric methods. Over time, the site has brought me into the planning stages more and more, and I have developed a greater awareness of the issues around DQOs, and consequently will try to have more input on them in future studies.

## 4. Conclusions

Environmental monitoring is obviously an important issue in our society, and it involves the bringing together of many scientific fields to properly assess sites that may present a threat to our environment. Statistical analyses are integral to any evaluation process where data are collected and used to make decisions, and environmental monitoring is no exception. In fact, analysis of environmental monitoring data often necessitates very complicated statistical methods. This is due to the limitations of machinery to measure chemical concentrations which often results in NDs in samples, the expense of collecting and measuring environment samples which leads to small sample sizes, the skewed distribution types that tend to best represent many environmental concentration populations, and commonly not being able to identify any distribution to model environmental data which then necessitates using nonparametric methods. To address such issues requires capable software and a high level of statistical competence. The EPA has implemented useful analysis tools in their ProUCL software, and provided a good resource for learning about the methodologies implemented in the ProUCL Technical Guide. However, ProUCL does have its limitations, largely due to funding. These limitations could be largely overcome if, instead of just providing these tools as a unified software product, the EPA would release the source code so that it could be implemented in other software environments and further built upon by researchers outside of the EPA and its contractors.

States have been delegated the responsibility to protect their own environment with guidance from the EPA. But to do this well requires adequate resources, and statistical expertise appears to be a greatly neglected resource by many, if not most, states. States have an ethical responsibility to both the sites that they monitor and the residents that they serve to ensure that adequate resources are provided to protect the state’s environment. In addition, state agencies overseeing environmental monitoring need to recognize the necessity for being able to conduct independent assessment of complicated statistical issues that may not be directly addressed by using EPA resources. There is, after all, very good reason that the ProUCL documents and various EPA guidance documents state that, project teams may want to consult a statistician.

## Figures and Tables

**Figure A1:**
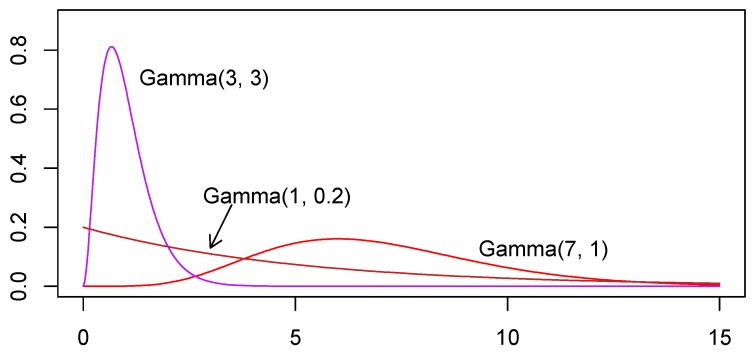
Probability density functions (pdfs) of the gamma distributions used in the simulations.

**Figure A2:**
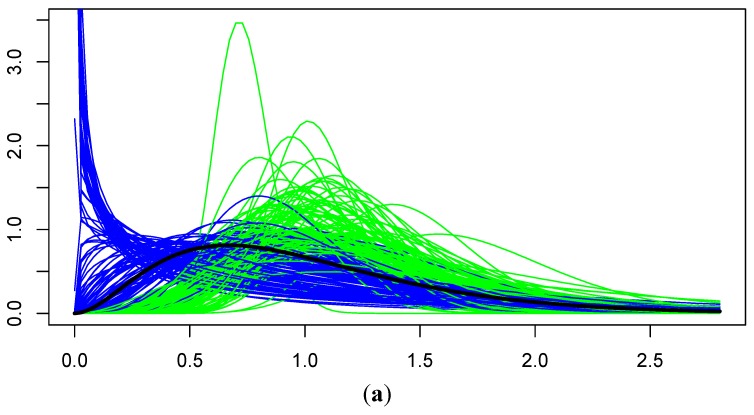

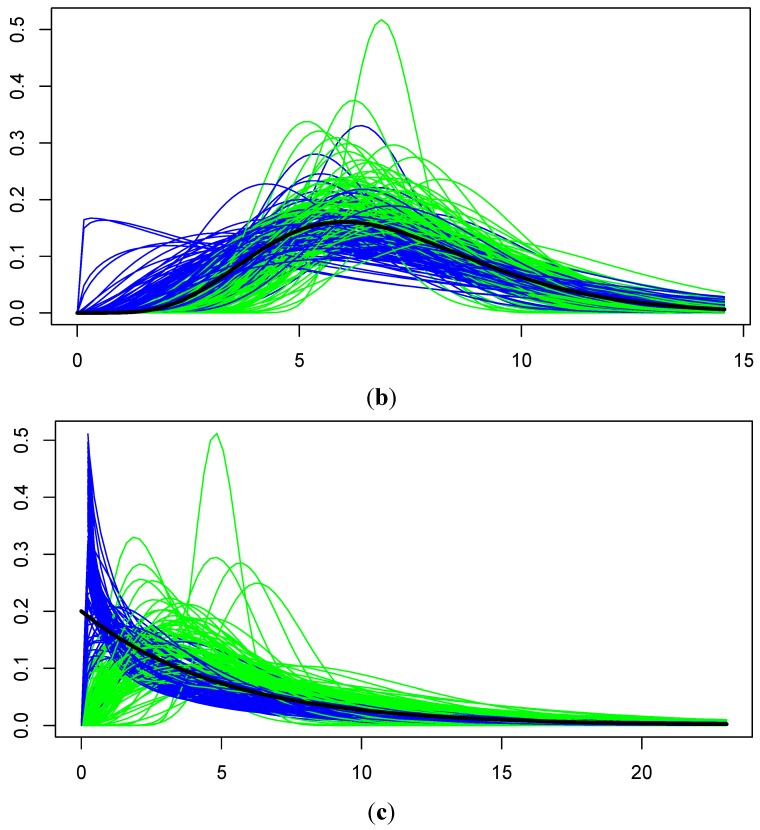
(**a**) Original Gamma(3, 3) pdf (black) for simulated data with *n* = 12 with an average of three NDs, and pdf’s estimated by the EM method (blue) and the GROS method (green); (**b**) Original Gamma(7, 1) pdf (black) for simulated data with *n* = 12 with an average of three NDs, and pdf’s estimated by the EM method (blue) and the GROS method (green); (**c**) Original Gamma(1, 0.2) pdf (black) for simulated data with *n* = 12 with an average of three NDs, and pdf’s estimated by the EM method (blue) and the GROS method (green).

## Appendix–Some Simulation Results

To demonstrate to the state agency the capability of the EM algorithm using gamma MLE estimation relative to the gamma ROS method, I presented them with simulation results for three gamma distributions having parameter sets (*i.e.*, shape and rate) that were fairly representative of the estimated gamma distributions encountered in the study. Namely,

1 Gamma (3, 3),
2 Gamma (7, 1), and
3 Gamma (1, 0.2).

Figure A1 shows the probability density functions (pdfs) for these distributions. From this plot one can see that the second distribution is approaching symmetry and somewhat approximates a normal distribution. Figure A2a–c show some results of the simulations implementing both the Gamma ROS method and the EM method. In the simulations, samples of size *n* = 12 were generated (since the simulations were targeted at the situation in the study we were working on) and NDs were inserted based on a DL that was selected in a manner that would produce an average of three NDs in a sample (since this was fairly common for the samples in the study). In Figure A2a–c, the pdf of the original gamma distribution from which simulation data were generated is shown with a heavy black curve. For each figure, 100 data sets were randomly generated and the EM method was used to estimate the gamma parameters-the corresponding pdfs are displayed in blue; and the gamma ROS method was used to estimate parameters for the same 100 data sets—these pdfs are displayed in green. In each of Figure A2a–c, the pdf’s obtained by the EM method generally approximates the original pdf more closely than the pdf’s obtained from the gamma ROS method.

Simulations using 10,000 iterations were also conducted to numerically compare the parameter estimates to the true parameter values and to compare the calculated 95%-confidence–95%-tolerance UTLs to the 95^th^ percentile of the original gamma distribution. Table A1 shows the results of these simulations.

**Table A1:** Simulation results showing mean distances and root mean squared deviations from the true parameter values. UTL measures are from the 95^th^ percentile of the true distribution. UTL % exceedance is the percentage of 95%–95% UTLs that exceeded the 95th percentile of the true distribution.

	Gamma (3, 3)		Gamma (7, 1)		Gamma (1, 0.2)	
	GROS	EM	GROS	EM	GROS	EM
Shape Mean Distance	6.384	−0.787	4.626	−0.770	1.765	−0.166
Shape Root-MS-Deviation	9.500	2.169	7.942	4.015	2.478	0.608
Rate Mean Distance	5.225	−0.761	0.614	−0.103	0.299	−0.022
Rate Root-MS-Deviation	8.572	2.119	1.114	0.569	0.522	0.129
UTL Mean Distance	2.451	1.915	4.059	5.074	22.89	14.97
UTL Root-MS-Deviation	3.245	2.319	5.274	6.337	27.06	18.26
UTL % Exceedance	93.88	96.30	92.73	95.29	95.49	95.37

From Table A1 we see that for the first and third distributions (which are more skewed) the EM method was more on target in estimating the gamma parameters by a factor of about 8–10, and by a factor of about 4–6 for the second distribution (which is more symmetric). For the first and third distributions the EM method had a smaller typical deviation (as measured by the root mean squared deviation) by a factor of about 4, and by a factor of about 2 for the second distribution. For the first and third distributions the EM method was more on target with its UTL by a factor of about 1.5, and had a smaller typical deviation also by a factor of about 1.4–1.5. For the second, more symmetric distribution, it was actually further off in its being on target and typical deviation, each having a factor of about 1.2 of the ROS UTL, but the ROS method fell a little short of obtaining the prescribed confidence of 95% for both this distribution and the first distribution, whereas the EM method obtained the prescribed confidence for each distribution, though never by much (0.29%–1.30%). In general, for this limited scenario, it appears that the EM method performs substantially better at parameter estimation and gets closer to the desired population percentile for all of the distributions examined, and is marginally better (factor of 1.5) at estimating the 95^th^ percentile with the UTL calculation when the distribution is not symmetric. For the symmetric distribution the EM’s UTL accuracy and precision is slightly lower (a factor of 1.2 of the ROS values), but achieved the 95% confidence while the ROS method fell a little short. All of the R code used to produce Figure A2a–c and to conduct the simulations presented in Table A1, including the starting random number seed, are available as a supplement to this paper on the journal’s web site.

These simulations were conducted with the specific goal of addressing issues with the environmental agency in the study that was being conducted. As this paper is a case study perspective, it is not the goal to present a more thorough simulation analysis here. Rather the goal is to illustrate the situation with the state agency. Of particular note is that these simulations are targeted at data that have only a single DL since the vast majority of the data in the case study were covered by this case (just over 80%). Additionally of note is that at the time of the discussion with the state agency, the KM method was not being considered because it was not part of the original proposal and the state agency had recommended we use an ROS method. Since the EM method uses an approach similar to the ROS method, we felt it fit within the specifications of the agency whereas the KM method clearly did not. However, it is also worth noting that an extension to the EM method has been developed that works well with multiple DLs but takes a different approach in dealing with the multiple DLs than the ROS method, and limited initial simulations appear to indicate that it performs well. Some adjustment in the algorithm has also been made that appears to improve the consistency in the parameter estimations (even for the single DL case) and in how well the estimated pdf curves track the original pdf (for example, in a plot similar to Figure A2a there are far fewer EM-estimated pdfs that asymptote on the Y-axis). This EM-based algorithm and its capabilities will be presented in a future paper once more simulations have been conducted.
